# Prognostic factors in patients with vulvar cancer treated with primary surgery: a single-center experience

**DOI:** 10.1186/s40064-016-1767-7

**Published:** 2016-02-18

**Authors:** Sayaka Imoto, Morihiko Inamine, Wataru Kudaka, Yutaka Nagai, Akihiko Wakayama, Tomoko Nakamoto, Takuma Ooyama, Yoichi Aoki

**Affiliations:** Department of Obstetrics and Gynecology, Graduate School of Medicine, University of the Ryukyus, 207 Uehara Nishihara, Okinawa, 903-0215 Japan

**Keywords:** Vulvar cancer, Primary surgery, Inguinal lymph node metastasis, Surgical margin

## Abstract

Vulvar cancer is a relatively rare disease. The aim of this study was to investigate prognostic factors in vulvar squamous cell carcinoma patients treated with primary surgery. Forty cases of vulvar squamous cell carcinoma treated with primary surgery were retrospectively analyzed. Overall survival (OS) and disease-specific survival (DSS) were calculated using the Kaplan–Meier method and prognostic factors were analyzed by multivariate analyses. The median age was 68 years. The FIGO stage distribution was as follows: 18 cases (45.0 %) in stage I, four cases (10.0 %) in stage II, 15 cases (37.5 %) in stage III, and three cases (7.5 %) in stage IV. A radical local excision was performed in 15 patients, and radical vulvectomy in 25 patients, and seven of these patients were treated with postoperative RT. The 5-year DSS rate was 72.6 %, and the 5-year OS rate was 70.3 %. Age and surgical margin ≤5 mm were independent prognostic factors for OS, and positive inguinal LN metastasis and surgical margin ≤5 mm were identified as independent prognostic factors for DSS. Complete radical excision is important regardless of operation mode. Adjuvant treatment should be considered for inguinal LN positive patients.

## Background

Vulvar cancer is a relatively rare disease, representing only 3–5 % of gynecologic malignancies. It typically affects older women aged 65–70 years, although the incidence among younger women is increasing. Around 90 % of vulvar cancers are squamous cell carcinomas. The standard treatment for early disease consists of radical local excision of the primary tumor with sentinel lymph node (LN) biopsy and/or inguinal lymphadenectomy. Advanced disease is often managed with a combination of radiation therapy (RT) and/or chemotherapy (Hacker 2005; Beller et al. 2006; Joura 2002). However, the appropriate treatment in each case should be selected by taking into account the age of the patient, tumor location, performance status (PS), and various complications. To date, there are limited data on treatment modalities, patterns of failure, and prognostic factors in Japanese patients with vulvar cancer. In the present study, we retrospectively reviewed the medical records for squamous cell carcinoma of the vulva cases treated with primary surgery in our department, and summarized prognostic factors and failure patterns.

## Patients and methods

Forty cases of vulvar squamous cell carcionma treated with primary surgery at the University of the Ryukyus Hospital from 1984 to 2012 were retrospectively analyzed. Clinicopathological characteristics (age, histologic type, tumor diameter, tumor marker, LN metastasis, distant metastasis, type of treatment, adverse effects of treatment, and site of recurrence) were surveyed. The International Federation of Gynecology and Obstetrics (FIGO) 2009 staging classification (Hacker 2009) was used. Past cases classified with the FIGO 1988 staging classification were reclassified based on the 2009 classification. Overall survival (OS) and disease-specific survival (DSS) were calculated using the Kaplan–Meier method, and log-rank tests were used to determine the significance. Prognostic factors for OS and DSS were analyzed using the Cox proportional hazard model, and risk factors for local–regional and distant recurrences were examined using logistic regression analysis. A *P* value <0.05 was considered statistically significant. All statistical analyses were performed using JMP v10 (SAS Institute Inc., Cary, NC).

All patients provided written informed consent before treatment. This retrospective study was conducted according to the principles stated in the 1964 Declaration of Helsinki with subsequent revisions and was approved by the Institutional Review Board of our university (#662) in June, 2014.

## Results

The median age was 68 years (range 37–90 years), and the median observation period was 62.5 months (range 4–353 months). The FIGO stage distribution was as follows: 18 cases (45.0 %) in stage I, four cases (10.0 %) in stage II, 15 cases (37.5 %) in stage III, and three cases (7.5 %) in stage IV. The median tumor size was 33 mm (range 8–105 mm). A radical local excision was performed in 15 patients, and radical vulvectomy in 25 patients, and seven of these patients were treated with postoperative RT. With regards to inguinal LN dissection, it was omitted in patients with suspicious stage IA, and unilateral dissection or sampling of LNs was performed in patients with small tumor in one side. During the follow-up period of 62.5 months, 19 patients showed no evidence of disease, and three were alive with disease. Seventeen patients (42.5 %) had recurrence, which was local–regional in three cases, distant in five cases, and both local–regional and distant in nine cases. Fourteen patients died of the vulvar cancer and four patients died of intercurrent disease (Table 1).

**Table 1:** Patient characteristics (N = 40)

Variables	
Age median (years) (range)	68 (37–90)
Stage (FIGO^a^ 2009)	
IA	6
IB	12
II	4
IIIA	7
IIIB	7
IIIC	1
IV	3
Tumor size (mm) (range)	33 (8–105)
Surgery	
Radical local excision	15
Radical vulvectomy	18
Radical vulvectomy + RT^b^	7
Recurrence sites	
Local–regional	3
Local–regional + distant	9
Distant	5
Prognosis	
NED^c^	19
AWD^d^	3
DOD^e^	14
DOID^f^	4
Follow up period (months)	62.5 (4–353)

^a^The International Federation of Obstetrics and Gynecology

^b^Radiation therapy

^c^No evidence of disease

^d^Alive with disease

^e^Died of disease

^f^Died of intercurrent disease

**Fig. 1:**
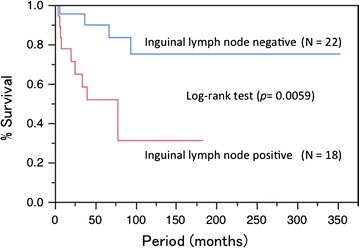
The 5-year disease-specific survival rate was 89.8 % in patients with positive inguinal lymph node and 51.9 % in patients with negative inguinal lymph node

**Fig. 2:**
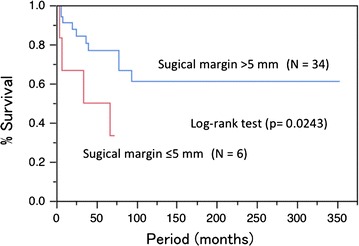
The 5-year disease-specific survival rate was 76.9 % in patients with surgical margin >5 mm and 50.0 % in patients with surgical margin ≤5 mm

The 5-year DSS rate was 72.6 %, and the 5-year OS rate was 70.3 %. The 5-year DSS and OS by each variable are shown in Table 2. The 5-year DSS rates were 82.5 % for stage IB, 100 % for stage II, 55.2 % for stage III, and 33.3 % for stage IV. The 5-year OS rates were 75.0 % for stage IB disease, 100 % for stage II, 55.2 % for stage III, and 33.3 % for stage IV. There were significant differences in survival among the FIGO stages. Positive inguinal LN metastasis and surgical margin ≤5 mm were significant and tumor size ≥4 cm was marginal for both DSS (Figs. 1, 2) and OS. Age ≥70 years was significant for OS but not for DSS. The 5-year DSS rates were 74.8 % for radical vulvectomy, and 68.3 % for radical local excision (*P* = 0.774), and the 5-year OS rates were 71.2 % for radical vulvectomy, and 68.3 % for radical local excision (*P* = 0.521). Therefore, there were no significant differences in survival among operation procedures.

**Table 2:** Univariate analysis for disease-free survival and overall survival

Variables	n	No. of DOD^d^	5y-DSS^e^ (%)	p value	No. of death	5y-OS^f^ (%)	p value
Age (years)							
70≤	23	7	51.0	0.146	11	46.4	0.0049
70≥	17	7	82.6		7	82.6	
Stage (FIGO 2009)							
Ia	6	0	100	0.0028	0	100.0	0.0093
Ib	17	2	82.5		5	75.0	
II	5	2	100		2	100.0	
III	16	7	55.2		8	55.2	
IV	4	3	33.3		3	33.3	
Tumor size							
4 cm≤	17	8	59.9	0.0577	9	54.9	0.0791
4 cm>	23	6	81.5		9	81.5	
Inguinal LN^a^ metastasis							
Negative	22	4	89.8	0.0059	7	85.4	0.0161
Positive	18	10	51.9		11	51.9	
Surgical resection margin							
>5 mm	34	10	76.9	0.0243	13	74.1	0.0066
≤5 mm	6	4	50.0		5	50.0	
Operation mode							
RLE^b^	15	5	68.3	0.774	7	68.3	0.521
RV^c^	25	9	74.8		11	71.2	

^a^Lymph node

^b^Radical local excision

^c^Radical vulvectomy

^d^Died of disease

^e^Disease-specific survival

^f^Overall survival

The prognostic factors for DSS and OS were analyzed using a Cox proportional hazard model. Age ≥70 years (*P* = 0.040) and surgical margin ≤5 mm (*P* = 0.028) were independent prognostic factors for OS, and positive inguinal LN metastasis and surgical margin ≤5 mm were identified as independent prognostic factors for DSS (Table 3). The predictive factors for local–regional and distant recurrence were analyzed using a logistic regression analysis. There was no significant predictive factor for local–regional and distant recurrences (data not shown).

**Table 3:** Cox proportional hazard model for disease-free survival and overall survival

	DSS^a^			OS^b^		
	HR^c^	95 % CI^d^	P value	HR^c^	95 % CI^d^	P value
Age ≥70 years	1.414	0.427–4.544	0.560	2.891	1.051–8.366	0.040
Tumor size ≥4 cm	1.417	0.4334–4.957	0.566	1.495	0.490–4.732	0.481
Inguinal LN^e^ metastasis	4.459	1.189–19.06	0.0264	2.639	0.825–8.570	0.101
Surgical margin ≤5 mm	4.640	1.128–17.57	0.0348	4.053	1.177–12.94	0.028

^a^Disease-specific survival

^b^Overall survival

^c^Hazard ratio

^d^Confidence interval

^e^Lymph node

## Discussion

In this study, the FIGO 2009 staging classification (Hacker 2009) was used, and cases prior to 2009 were reclassified based on this classification. The performance of the revised staging system had been assessed in a number of studies; however, the results are conflicting (van der Steen et al. 2010; Tabbaa et al. 2012). Looking at OS and DSS based on the FIGO stage, the classification gave a good spread of prognostic groupings. Tumor size was not an independent prognostic factor. Inguinal LN metastasis, and the number, size, and morphology of the positive nodes are taken into account in the classification. LN metastasis is a good prognostic marker that provides a clear distribution across tumor stages, although we could not apply it to stages IIIA, IIIB, and IIIC because of the small number of patients in the present study. The FIGO 2009 staging classification seems to allow better prognostic discrimination between stages and less heterogeneity within stages than the earlier version.

In most cases, radical vulvectomy with inguinofemoral lymphadenectomy should be considered as an appropriate treatment for vulvar cancer in the 70s and early 80s. However, to reduce surgical morbidity, radical local excision of the primary tumor has generally replaced radical vulvectomy. Previous studies reported no significant difference in prognosis for this approach, even when compared with extensive surgery (De Hullu et al. 2002; Stehman et al. 1992). Also, in our patients, no significant difference in prognosis was observed among the operative procedures. Radical local excision of the primary tumor has generally instead of radical vulvectomy is appropriate to reduce surgical morbidity.

In the multivariate analysis in the present study, age ≥70 years and close surgical resection margin were independent prognostic factors for OS, but not for DSS. In our study population, the fact that four patients died of intercurrent disease and all patients were aged ≥70 years. Furthermore, under treatment of elderly patients was observed, such as postoperative radiotherapy was omitted in six of 8 patients with positive groin nodes and two of three patients with close surgical margin. These points may have affected OS. A surgical resection margin <8 mm has been reported to be associated with a high recurrence rate (Heaps et al. 1990). Cases with a surgical margin ≤5 mm have a high local recurrence rate, but radiation with a dose ≥56 Gy may decrease the risk of vulvar recurrence (Viswanathan et al. 2013). Therefore, pathologic margin distance is an important predictor of local vulvar recurrence. Chan et al. (2007) suggested that a ≥8 mm pathologic margin clearance leads to a high rate of local–regional control. Our data were consistent with the findings of the previous studies.

In our study, 18 patients had positive inguinal LNs. Seven of these patients underwent postoperative RT, whereas 11 were treated with surgical resection only. Of those 18 patients, 11 had recurrence, including 3 of the 7 patients who underwent postoperative RT, and eight of the 11 patients were treated with surgical resection only. The patients who received no radiation included higher age (n = 6), stage IIIA (n = 3) and unknown (n = 2). Only local–regional recurrence was observed in one case, only distant recurrence in four, and both local–regional and distant recurrences in six cases. An increased risk of recurrence has been described in patients with LN metastases, large primary tumors, deep invasion, lymphovascular invasion and close surgical margins (Heaps et al. 1990; Binder et al. 1990; Burger et al. 1995; Woelber et al. 2009). RT is playing an increasing role in the management of patients with carcinoma of the vulva, in combination with radical local excision. Post radical vulvectomy RT in locally advanced tumors improves tumor control at the primary site and in the regional lymphatics compared with surgery alone (Perez et al. 1998). RT alone or in combination with LN dissection is highly effective in preventing inguinal node recurrence in patients with squamous cell carcinoma of the vulva (Katz et al. 2003). Adjuvant groin and pelvic RT is the standard of care for node-positive vulvar squamous cell carcinoma for patients with two or more involved LNs, extracapsular extension, or inadequate LN dissection based on Gynecologic Oncology Group (GOG) 37 (Homesley et al. 1986). Because of a limited number of patients with only one LN involved, adequate power was not reached to determine the benefit of RT (Kunos et al. 2009). A Surveillance, Epidemiology, and End Result (SEER) analysis indicated that for single inguinal LN involvement, adjuvant RT improved disease-specific survival and increased OS if <12 LNs were removed (Parthasarathy et al. 2006). However, not all studies of adjuvant radiation have supported these results (Groenen et al. 2010; Fons et al. 2009).

While the addition of concurrent chemotherapy to RT has increased over the past decade for advanced vulvar cancers (Han et al. 2000; Moore et al. 2012), the impact in the adjuvant setting is currently unknown. Recently, a population-based analysis using the National Cancer Data Base (NCDB) showed that adjuvant chemotherapy resulted in a significant reduction in mortality risk for node-positive vulvar cancer patients who received adjuvant RT, reinforcing the use of adjuvant chemotherapy to improve outcomes in this high-risk subset (Gill et al. 2015). Because our patients had high frequency of distant failure, we may consider adjuvant chemotherapy concurrent with RT.

Strength of our study is past cases classified with the FIGO 1988 staging classification were reclassified based on the 2009 classification. We have shown a good prognostic distribution of FIGO 2009 classification. Weakness is this analysis is retrospective in a small number of patients from one center over a relatively long period of time.

In conclusion, we retrospectively analyzed 40 patients with squamous cell carcinoma of the vulva treated with primary surgery. The important prognostic factors were inguinal LN metastasis and close surgical resection margin. Complete radical excision is important regardless of operation mode. Adjuvant treatment should be considered for inguinal LN positive patients.

## References

[CR1] Beller U, Quinn MA, Benedet JL (2006). Carcinoma of the vulva: FIGO 26th annual report on the results of treatment in gynecological cancer. Int J Gynaecol Obstet.

[CR2] Binder SW, Huang I, Fu YS (1990). Risk factors for the development of lymph node metastasis in vulvar squamous cell carcinoma. Gynecol Oncol.

[CR3] Burger MP, Hollema H, Emanuels AG (1995). The importance of the groin node status for the survival of T1 and T2 vulvar carcinoma patients. Gynecol Oncol.

[CR4] Chan JK, Sugiyama V, Pham H (2007). Margin distance and other clinico-pathologic prognostic factors in vulvar carcinoma: a multivariate analysis. Gynecol Oncol.

[CR5] De Hullu JA, Hollema H, Lolkema S (2002). Vulvar carcinoma. The price of less radical surgery. Cancer.

[CR6] Fons G, Groenen SM, Oonk MH (2009). Adjuvant radiotherapy in patients with vulvar cancer and one intra capsular lymph node metastasis is not beneficial. Gynecol Oncol.

[CR7] Gill BS, Bernard ME, Lin JF (2015). Impact of adjuvant chemotherapy with radiation for node-positive vulvar cancer: a National Cancer Data Base (NCDB) analysis. Gynecol Oncol.

[CR8] Groenen SM, Timmers PJ, Burger CW (2010). Recurrence rate in vulvar carcinoma in relation to pathological margin distance. Int J Gynecol Cancer.

[CR9] Hacker NF, Berek JS, Hacker NF (2005). Vulvar cancer. Practical gynecologic oncology.

[CR10] Hacker NF (2009). Revised FIGO staging for carcinoma of the vulva. Int J Gynecol Obstet.

[CR11] Han SC, Kim DH, Higgins SA (2000). Chemoradiation as primary or adjuvant treatment for locally advanced carcinoma of the vulva. Int J Radiat Oncol Biol Phys.

[CR12] Heaps JM, Fu YS, Montz FJ (1990). Surgical-pathologic variables predictive of local recurrence in squamous cell carcinoma of the vulva. Gynecol Oncol.

[CR13] Homesley HD, Bundy BN, Sedlis A (1986). Radiation therapy versus pelvic node resection for carcinoma of the vulva with positive groin nodes. Obstet Gynecol.

[CR14] Joura EA (2002). Epidemiology, diagnosis and treatment of vulvar intraepithelial neoplasia. Curr Opin Obstet Gynecol.

[CR15] Katz A, Eifel PJ, Jhingran A (2003). The role of radiation therapy in preventing regional recurrences of invasive squamous cell carcinoma of the vulva. Int J Radiat Oncol Biol Phys.

[CR16] Kunos C, Simpkins F, Gibbons H (2009). Radiation therapy compared with pelvic node resection for node-positive vulvar cancer: a randomized controlled trial. Obstet Gynecol.

[CR17] Moore DH, Ali S, Koh WJ (2012). A phase II trial of radiation therapy and weekly cisplatin chemotherapy for the treatment of locally-advanced squamous cell carcinoma of the vulva: a gynecologic oncology group study. Gynecol Oncol.

[CR18] Parthasarathy A, Cheung MK, Osann K (2006). The benefit of adjuvant radiation therapy in single-node-positive squamous cell vulvar carcinoma. Gynecol Oncol.

[CR19] Perez CA, Grigsby PW, Chao C (1998). Irradiation in carcinoma of the vulva: factors affecting outcome. Int J Radiat Oncol Biol Phys.

[CR20] Stehman FB, Bundy BN, Dvoretsky PM (1992). Early stage I carcinoma of the vulva treated with ipsilateral superficial inguinal lymphadenectomy and modified radical hemivulvectomy: a prospective study of the Gynecologic Oncology Group. Obstet Gynecol.

[CR21] Tabbaa ZM, Gonzalez J, Sznurkowski JJ (2012). Impact of the new FIGO 2009 staging classification for vulvar cancer on prognosis and stage distribution. Gynecol Oncol.

[CR22] van der Steen S, de Nieuwenhof HP, Massuger L (2010). New FIGO staging system of vulvar cancer indeed provides a better reflection of prognosis. Gynecol Oncol.

[CR23] Viswanathan AN, Pinto AP, Schultz D (2013). Relationship of margin status and radiation dose to recurrence in post-operative vulvar carcinoma. Gynecol Oncol.

[CR24] Woelber L, Mahner S, Voelker K (2009). Clinicopathological prognostic factors and patterns of recurrence in vulvar cancer. Anticancer Res.

